# Multiple *Salmonella*-pathogenicity island 2 effectors are required to facilitate bacterial establishment of its intracellular niche and virulence

**DOI:** 10.1371/journal.pone.0235020

**Published:** 2020-06-25

**Authors:** Katelyn Knuff-Janzen, Audrey Tupin, Sophie Yurist-Doutsch, Jennifer L. Rowland, B. Brett Finlay

**Affiliations:** 1 Michael Smith Laboratories, University of British Columbia, Vancouver, British Columbia, Canada; 2 Department of Microbiology & Immunology, University of British Columbia, Vancouver, British Columbia, Canada; 3 Department of Biochemistry and Molecular Biology, University of British Columbia, Vancouver, British Columbia, Canada; East Carolina University Brody School of Medicine, UNITED STATES

## Abstract

The pathogenesis of *Salmonella* Typhimurium depends on the bacterium’s ability to survive and replicate within host cells. The formation and maintenance of a unique membrane-bound compartment, termed the *Salmonella*-containing vacuole (SCV), is essential for *S*. Typhimurium pathogenesis. SCV-bound *S*. Typhimurium induces formation of filamentous tubules that radiate outwards from the SCV, termed *Salmonella*-induced filaments (SIFs). SIF formation is concomitant with the onset of replication within host epithelial cells. SIF biogenesis, formation and maintenance of the SCV, and the intracellular positioning of the SCV within the host cell requires translocation of bacterial proteins (effectors) into the host cell. Effectors secreted by the type III secretion system encoded on *Salmonella* pathogenicity island 2 (T3SS2) function to interfere with host cellular processes and promote both intracellular survival and replication of *S*. Typhimurium. Seven T3SS2-secreted effectors, SifA, SopD2, PipB2, SteA, SseJ, SseF, and SseG have previously been implicated to play complementary, redundant, and/or antagonistic roles with respect to SIF biogenesis, intracellular positioning of the SCV, and SCV membrane dynamics modulation during infection. We undertook a systematic study to delineate the contribution of each effector to these processes by (i) deleting all seven of these effectors in a single *S*. Typhimurium strain; and (ii) deleting combinations of multiple effectors based on putative effector function. Using this deletion mutant library, we show that each of SIF biogenesis, intracellular SCV localization, intramacrophage replication, colonization, and virulence depends on the activities of multiple effectors. Together, our data demonstrates the complex interplay between these seven effectors and highlights the necessity to study T3SS2-secreted effectors as groups, rather than studies of individual effectors.

## Introduction

*Salmonella enterica* serovar Typhimurium (*S*. Typhimurium) is a Gram-negative foodborne pathogen that commonly causes non-typhoidal salmonellosis (gastroenteritis) in humans. The success of *Salmonella* as an intracellular pathogen is largely due to its ability to evade host defense mechanisms by invading and residing within intestinal epithelial cells and phagocytic cells of the host [1,2]. Upon invasion of the host cell, *S*. Typhimurium resides within a unique compartment called the *Salmonella*-containing vacuole (SCV). Formation and maintenance of the SCV during infection is critical to survival within phagocytes and plays an important role in promoting *S*. Typhimurium replication in non-phagocytic cells [3].

The SCV, while a unique compartment distinct from host organelles, initially exhibits features similar to maturing endosomes. Upon invasion of the host cell, the nascent SCV (early SCV) carries the same membrane markers that characterize early endosomes such as EEA1 and transferrin receptor [4]. Subsequent SCV maturation occurs through a series of controlled selective sequential interactions with the host’s endocytic pathway [4,5]. During maturation, the SCV—like late endosomes—accumulates lysosomal glycoproteins such as lysosomal associated membrane proteins (LAMP) 1 and 2 within the SCV membrane and the SCV lumen acidifies. However, unlike late endosomes, the SCV does not fully mature into a lysosome owing to manipulation of the host cell by intravacuolar *S*. Typhimurium [6–9].

*S*. Typhimurium possesses two distinct type III secretion systems (T3SSs) encoded on *Salmonella* pathogenicity islands 1 and 2 (T3SS1 and T3SS2 respectively). These two T3SSs, along with additional virulence factors, allow *S*. Typhimurium to invade, survive, and replicate within host cells [2]. Whereas the T3SS1-secreted effectors are primarily associated with facilitating invasion of non-phagocytic cells and initial formation of the early SCV, the T3SS2-secreted effectors generally function to promote replication within both phagocytic and non-phagocytic cells [10–12]. The T3SS2-secreted effectors exert a wide variety of functions during infection including, but not limited to, maintaining the SCV membrane, regulating intracellular SCV positioning, and forming the membranous filament-like extensions that radiate outwards from the SCV, termed *Salmonella*-induced filaments (SIFs) [13]. SIF biogenesis begins at 4–6 hours post-infection, concomitant with the onset of intercellular bacterial replication in human epithelial cells [14–16]. SIFs are thought to play a number of important roles during infection including nutrient acquisition from the host and cell-to-cell transfer [17–20].

A subset of seven T3SS2-secreted effectors have been shown to play a role in SIF biogenesis, intracellular positioning of the SCV, and in controlling SCV membrane dynamics. These effectors of interest include: SifA, SseF, SseG, SteA, PipB2, SopD2, and SseJ [10,11,21–23]. The single deletion mutant of each of these seven effectors results in attenuation of virulence in the mouse model of systemic infection [13,22,24–27] and all but PipB2 contribute to survival in mouse macrophages [10]. These data highlight the importance of these effectors in both *in vitro* and *in vivo* infection models. The precise function of the seven effectors of interest is known for some but unclear for others, and their contribution to formation of the intracellular replication niche remains ambiguous. Each of SifA, SseF, SseG, SteA, PipB2, SopD2, and SseJ contribute to at least one, if not several of the following roles during infection: SIF biogenesis, precise intracellular positioning of the SCV, SCV membrane stability, SCV membrane modification, microtubule recruitment, and/or regulation of microtubule motor activity at the SCV membrane. The effectors’ overlapping roles during infection make it difficult to determine precise effector function when studying a single effector at a time. Increasing evidence suggests that T3SS2-secreted effectors cooperate to facilitate the interaction of *S*. Typhimurium with host cell machinery, leading to events such as SIF biogenesis and SCV movement [28–31].

In this study, we systematically constructed a *S*. Typhimurium SL1344-based strain that lacks all seven of our effectors of interest, as well as multiple effector deletion combinations. We show that LAMP1^+^-tubule (SIF) extension is not exclusively driven by SifA, but rather, likely requires the activity of other effectors. We also demonstrate that LAMP1^+^-tubule extension, intracellular positioning, intramacrophage replication, and replication *in vivo* all require the action of multiple effectors. One effector alone does not solely mediate a single process.

## Results

### Construction of multi-effector deletion mutants

Through an extensive literature search we identified seven effectors of interest implicated in SIF biogenesis, SCV membrane maintenance, and intracellular SCV localization. These effectors include SseF, SseG, SteA, PipB2, SopD2, SseJ, and SifA (summarized in [32]). In order to address the redundancy and coordination of these effectors, we constructed a series of effector-deletion mutants (see Table 1) in the wild type *S*. Typhimurium SL1344 genetic background. We generated multiple-effector deletion mutants of the seven effectors of interest in a stepwise manner, using a suicide vector-based approach and homologous recombination [33], to generate a strain lacking all seven effectors, as well as specific combinations of effectors (see Table 1). The Δ*sseFG* strain does not express the two T3SS2-secreted effectors SseF or SseG encoded by the genes *sseF* and *sseG*, respectively, which are a part of the *sseABCDEFG* operon [34]. Within epithelial cells the replication, SCV localization, and appearance and frequency of SIFs in the Δ*sseF* single-effector deletion mutant very closely resembles both the Δ*sseG* single-effector deletion mutant and the Δ*sseFG* double deletion mutant, likely owing to the functional link between the two effectors [35,36]. We therefore consider the double-deletion mutant, Δ*sseFG*, effectively as a single-effector deletion mutant. Deleting one, or multiple coding regions for T3SS2-secreted effectors does not significantly impair the fitness of the effector deletion strains in LB broth (S1 Fig).

**Table 1:** Bacterial strains used in the study.

***Escherichia coli* Strains**				
		**Strain Designation**	**Relevant Characteristics/Genotype**	**Source/Reference**
		MC1061*λpir*	*hsdR mcrB araD139* Δ*(araABC-leu)7679* Δ*lacX74 gal1 galK rpsL thiλpir*	[37]
		MFD*pir*	*MG1655 RP4-2-TC*::*[*Δ*Mu1*::*aac(3)IV-*Δ*aphA-*Δ*nic35-*Δ*Mu2*::*zeo]* Δ*dapA*::*(erm-pir)* Δ*recA*	[38]
		DH10B	*F*^*-*^ *araDJ39* Δ*(ara*, *leu)7697* Δ*lacX74 galU galK rpsL deoR ɸ80dlacZ*Δ*M15 endAI nupG recAl mcrA* Δ*(mrr hsdRMS mcrBC)*	[39]
***Salmonella* Typhimurium Strains**				
		**Strain Designation**	**Relevant Characteristics/Genotype**	**Source/Reference**
		SL1344	Wild type stain, *hisG*	[40]
Single-effector deletion mutants		Δ*steA*	SL1344Δ*steA*	This study
		Δ*pipB2*	SL1344Δ*pipB2*	This study
		Δ*sopD2*	SL1344Δ*sopD2*	This study
		Δ*sseJ*	SL1344Δ*sseJ*	This study
		Δ*sifA*	SL1344Δ*sifA*	This study
		Δ*ssaR*	SL1344Δ*ssaR*	[41]
		Δ*sseFG*	SL1344Δ*sseF*Δ*sseG*	This study
Multi-effector deletion mutants	Sequential-effector deletion mutants	Δ*sseFG*Δ*steA*	SL1344Δ*sseF*Δ*sseG*Δ*steA*	This study
		Δ*sseFG*Δ*steA*Δ*pipB2*	SL1344Δ*sseF*Δ*sseG*Δ*steA*Δ*pipB2*	This study
		Δ*sseFG*Δ*steA*Δ*pipB2*Δ*sopD2*	SL1344Δ*sseF*Δ*sseG*Δ*steA*Δ*pipB2*Δ*sopD2*	This study
		Δ*sseFG*Δ*steA*Δ*pipB2*Δ*sopD2*Δ*sseJ*	SL1344Δ*sseF*Δ*sseG*Δ*steA*Δ*pipB2*Δ*sopD2*Δ*sseJ*	This study
		Δ*sseFG*Δ*steA*Δ*pipB2*Δ*sopD2*Δ*sseJ*Δ*sifA*	SL1344Δ*sseF*Δ*sseG*Δ*steA*Δ*pipB2*Δ*sopD2*Δ*sseJ*Δ*sifA*	This study
		Δ*sseFG*Δ*sseJ*	SL1344Δ*sseF*Δ*sseG*Δ*sseJ*	This study
		Δ*sseFG*Δ*sopD2*	SL1344Δ*sseF*Δ*sseG*Δ*sopD2*	This study
		Δ*sifA*Δ*sseJ*	SL1344 *sifA*Δ*sseJ*	This study
		Δ*sifA*Δ*sopD2*	SL1344Δ*sifA*Δ*sopD2*	This study
		Δ*sifA*Δ*sseJ*Δ*steA*	SL1344Δ*sifA*Δ*sseJ*Δ*steA*	This study
		Δ*sifA*Δ*sseJ*Δ*sopD2*	SL1344Δ*sifA*Δ*sseJ*Δ*sopD2*	This study

### Formation of LAMP1-positive tubules is dependent on multiple effectors

We analyzed the contribution of each of the effectors of interest to the formation of LAMP1-positive (LAMP1^+^) tubules that radiate outwards from the SCV. SIFs, the first of the *Salmonella*-induced tubules to be described [15,42,43], are identified by the presence of the host membrane protein LAMP1 within their membranes [23,44]. HeLa human epithelial cells were infected with the various effector deletion mutants and evaluated for the frequency of SIF formation in infected cells. Infected HeLa cells were fixed at 8 hours post-infection and immunolabeled with an anti-LAMP1 antibody to label LAMP1-positive compartments (SCVs) and tubules (SIFs) and anti-*Salmonella* antibody to label intracellular *S*. Typhimurium. Labeled cells were analyzed by indirect immunofluorescence microscopy. SIFs are, by definition, LAMP1^+^-tubules comprised of an inner and outer membrane that extend outwards from the SCV [43]. As we are unable to evaluate whether the SIFs observed are single- or double-membraned using this methodology, we will hereafter refer to them as LAMP1^+^-tubules.

**Fig 1:**
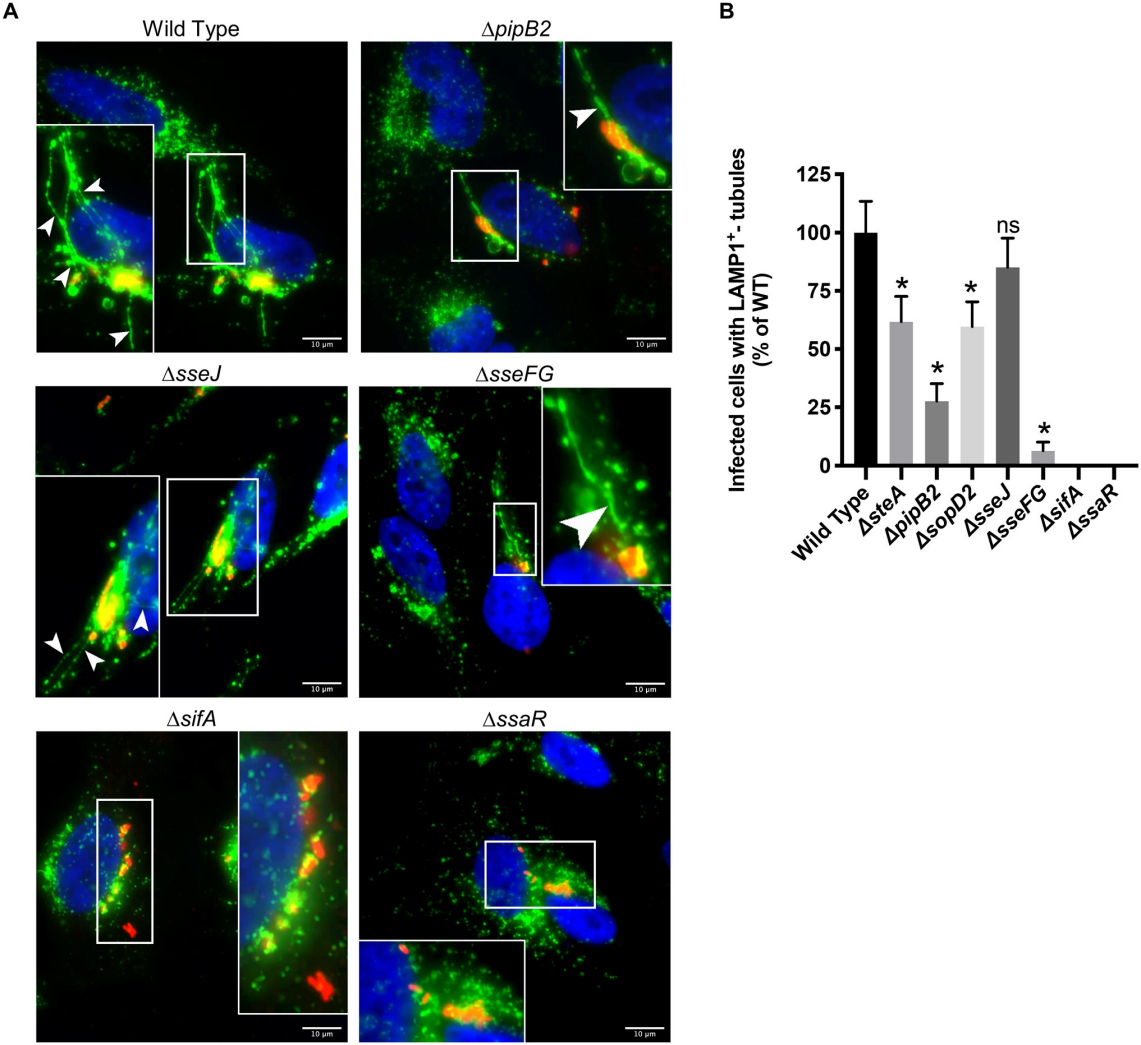
LAMP1^+^-tubule extension from single deletion mutants. **(A)** Comparison of frequency of LAMP1^+^-tubule formation of WT and isogenic single-effector deletion mutants in HeLa cells after 8 hours of infection. Cells were immunostained for *Salmonella* (red) and LAMP1 (green), and the nucleus was stained with DAPI (blue). Representative images of select strains are shown. The white boxes indicate zoomed-in region in inset. Arrowheads indicate LAMP1^+^-tubules. Scale Bar = 10 *μ*m. **(B)** Quantification of LAMP1^+^-tubule frequency in HeLa cells infected with the single deletion mutants for 8 hours. The average frequency of infected cells with LAMP1^+^-tubules relative to wild type infected cells ± standard error of the mean for three separate experiments is shown (*n* = 3). At least 100 infected cells per strain were blindly analyzed in each experiment. An asterisk indicates a significant difference between the indicated mutant strain LAMP1^+^-tubule frequency and the corresponding WT LAMP1^+^-tubule frequency (*p* < 0.02) as determined by Kruskal-Wallis one-way ANOVA with Dunn’s multiple comparison post-test.

Cells infected with the wild type strain and single-deletion mutants (Fig 1) exhibit LAMP1^+^-tubule formation consistent in both morphology and frequency to previous reports [14,15,28,36,43,45,46]. We observed “bulky” LAMP1^+^-tubules extending outwards from the SCV of Δ*pipB2* infected cells, consistent with previous reports [46] (Fig 1A). All single-effector deletion mutant strains, with the exception of Δ*sseJ*, had significantly fewer LAMP1^+^-tubule-positive infected cells relative to wild type, while the Δ*sifA* and Δ*ssaR* strains failed to form LAMP1^+^-tubules.

**Fig 2:**
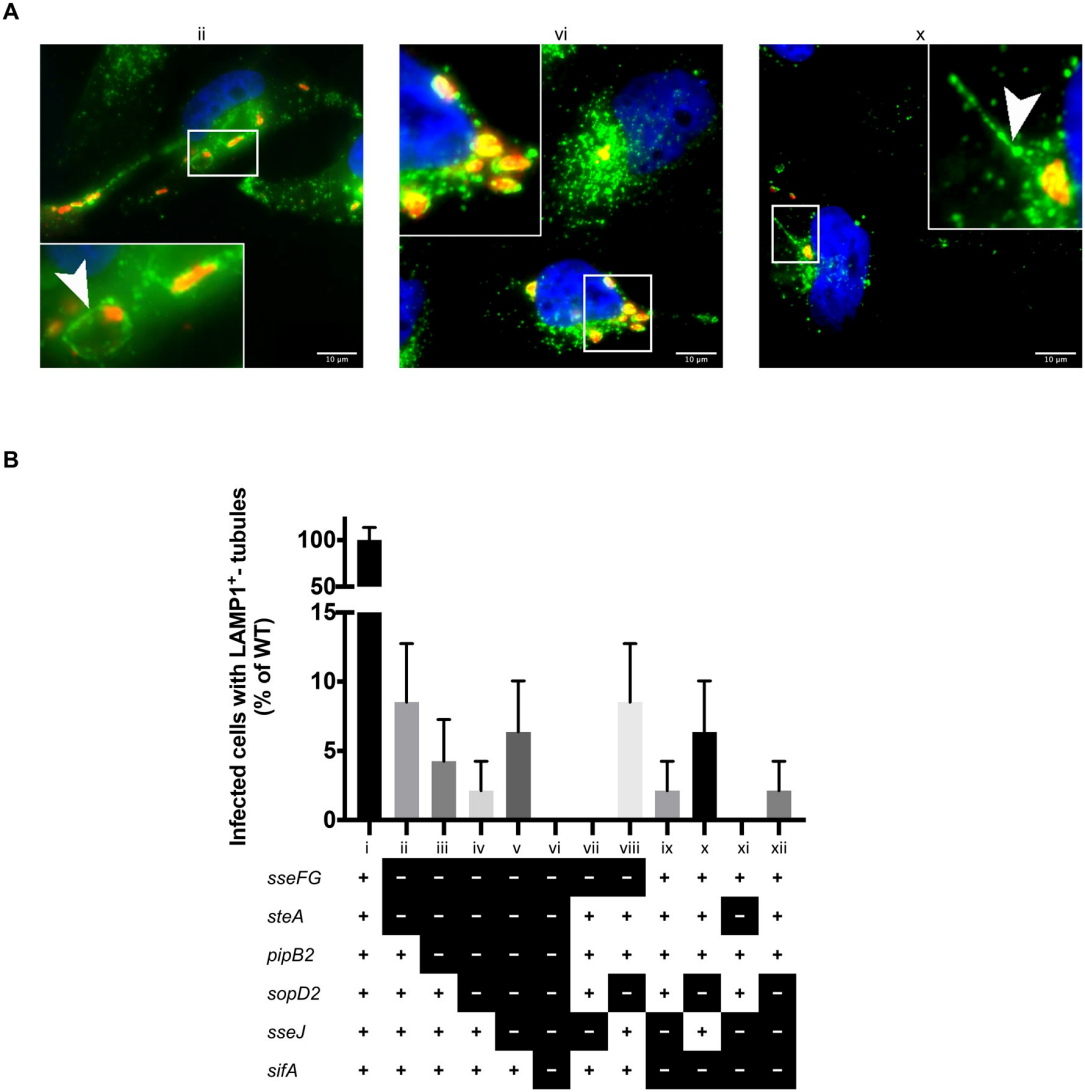
LAMP1^+^-tubule extension from the SCV results from the actions of several effectors. **(A)** Comparison of frequency of LAMP1^+^-tubule formation of WT and isogenic multiple-effector deletion mutants in HeLa cells after 8 hours of infection. Cells were fixed at 8 hours post-infection, immunostained, and analyzed as described in the legend of Fig 1. Representative images of select strains are shown. Strain designation (ii, vi, and x) corresponds to strains described in the legend of (B). **(B)** Quantification of LAMP1^+^-tubule frequency in HeLa cells infected with the multiple-effector deletion mutants for 8 hours. LAMP1^+^-tubule frequency was quantified and analyzed as described in the legend of Fig 1. Strain legend: “+” = gene present, “-” = gene deleted. A “+” for all genes indicates wild type (strain i). Results analyzed by a Kruskal-Wallis test with Dunn’s correction for multiple comparisons. All LAMP1^+^-tubule frequencies in the multiple-deletion mutant strains were significantly different from wild type, however there was no significance between the multiple-deletion mutants themselves.

Cells infected with the multiple-effector deletion mutant strains (Table 1) exhibit a dramatic decrease in the frequency of LAMP1^+^-tubule formation relative to both the wild type strain (Fig 2B) and the corresponding single-effector deletion mutants (Fig 1B). The sequential-effector deletion mutants (Fig 2B, strains ii-vi)—a subset of the multiple-effector deletion mutants—were found to have LAMP1^+^-tubules extending outwards from intracellular *Salmonella* in 2–8% of infected cells relative to wild type infected cells (Fig 2, strain i). The frequency of LAMP1^+^-tubule-positive infected cells was not statistically different between the sequential-effector deletion mutants. The sequential deletion of effectors does not dramatically reduce LAMP1^+^-tubule frequency (*i*.*e*. Δ*sseFGΔsteA* vs. Δ*sseFGΔsteAΔpipB2* vs. Δ*sseFGΔsteAΔpipB2ΔsopD2*). The sequential-effector deletion mutants Δ*sseFGΔsteA*, Δ*sseFGΔsteAΔpipB2*, Δ*sseFGΔsteAΔpipB2ΔsopD2*, and Δ*sseFGΔsteAΔpipB2ΔsopD2ΔsseJ* (Fig 2, strains ii-v) can all induce formation LAMP1^+^-tubules and only the sequential-effector deletion mutant with all seven effectors deleted (Δ*sseFGΔsteAΔpipB2ΔsopD2ΔsseJΔsifA*) fails to induce LAMP1^+^-tubules. This is consistent with previous evidence suggesting that SifA plays a major role in inducing LAMP1^+^-tubules [47] as all the sequential-effector deletion mutants are able to induce LAMP1^+^-tubule formation except for the strain with the *sifA* deletion.

Infection of HeLa cells with the remainder of the multiple-effector deletion mutants with different combinations of deleted effectors reveals that the mechanism of LAMP1^+^-tubule extension is indeed very intricate. The strain Δ*sseFGΔsseJ* (Fig 2, strain vii) is unable to form LAMP1^+^-tubules even though this strain has SifA. This contrasts with the above results from the sequential-effector deletion mutants suggesting that mutant strains can form LAMP1^+^-tubules so long as *sifA* was not deleted. Unlike Δ*sseFGΔsseJ*, the strain Δ*sseFGΔsopD2* (Fig 2, strain viii) was able to form LAMP1^+^-tubules. This may suggest an interaction or coordinated roles between SseJ and SseF/G that facilitates LAMP1^+^-tubule extension. For example, SseF/G may require the activity of SseJ in order to induce LAMP1^+^-tubulation which could explain the presence of LAMP1^+^-tubules in Δ*sseFGΔsopD2* infected cells but not Δ*sseFGΔsseJ* infected cells. Further studies are required to delve deeper into the reasons behind the formation of LAMP1^+^-tubules in some multiple-effector deletion strains and not in others.

The ability to form SIFs (LAMP1^+^-tubules) has long been thought to be heavily dependent on SifA as ectopic expression of SifA in HeLa cells induces LAMP1^+^-tubule formation [24,47]. We observed LAMP1^+^-tubules radiating outwards from the SCV in cells infected with both Δ*sifAΔsseJ* and Δ*sifAΔsopD2* double deletion mutants (Fig 2, strains ix and x, respectively), albeit at a very low frequency. Both strains lack the gene encoding *sifA*, yet they retain the ability to form LAMP1^+^-tubules. Intriguingly, we did not observe any LAMP1^+^ tubules in the Δ*sifAΔsseJΔsopD2* triple-effector deletion mutant (Fig 2, strain xi). This may indicate a required sequential or coordinated actions of SifA, SopD2, and SseJ, in order to extend LAMP1^+^-tubules.

### Intracellular localization of *S*. Typhimurium is modulated by multiple effectors

**Fig 3:**
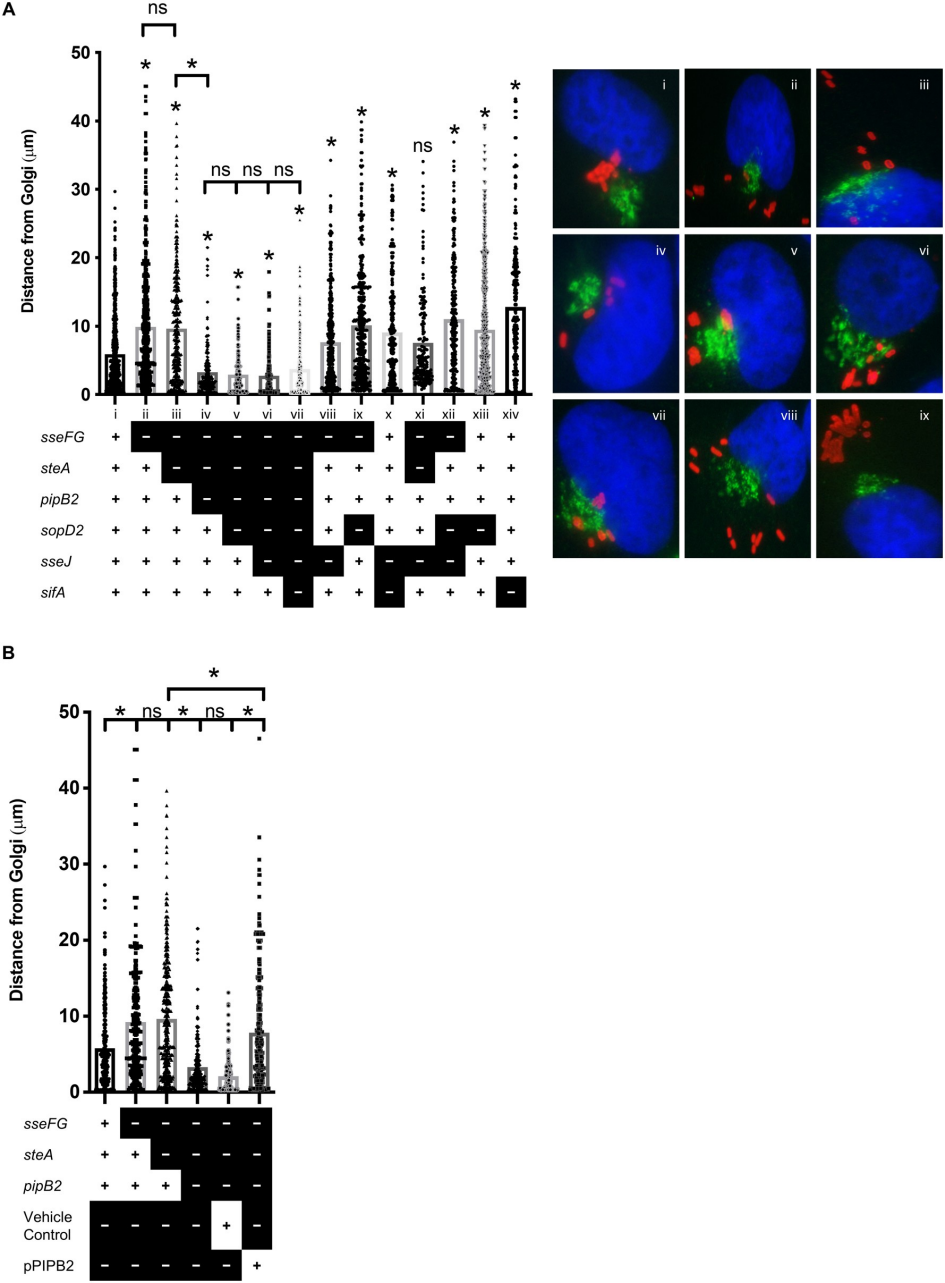
Multiple effectors drive SCV movement away from the Golgi complex. HeLa cells were infected with the indicated *S*. Typhimurium strains for 8 hours, fixed, and immunostained for *Salmonella* (red) and Golgin-97 (green), and the nucleus was stained with DAPI (blue). **(A)** Quantification of *S*. Typhimurium position relative to the Golgi. The distances from the center of individual bacteria to the nearest edge of the Golgi complex was measured in infected cells. Strain legend: “+” = gene present, “-” = gene deleted. All data points are shown to accurately indicate the spread of the data. The averages for three separate experiments are shown (*n* = 3). An asterisk indicates a significant difference (*p* < 0.003) between the indicated mutant strain and the corresponding WT strain or other strain if indicated by 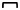 as determined by a Kruskal-Wallis one-way ANOVA with Dunn’s multiple comparison post-test. ns = not significant. **(B)** Select representative images used to enumerate distances in (A). **(C)** SL1344 strain Δ*sseFG*Δ*steA*Δ*pipB2* was complemented with a low-copy plasmid expressing a functional copy of PipB2. HeLa cells were infected, fixed, stained, and analyzed as described in (A).

Intracellular *S*. Typhimurium typically forms microcolonies near the microtubule-organizing center and Golgi-complex several hours post-infection in infected epithelial cells [35,48–50]. The T3SS2-secreted effectors SseF, SseG, SifA, PipB2, and SteA have been individually implicated in SCV localization during infection [35,48–53]. We used our library of multiple-effector deletion mutants to examine the effect of multiple effector deletions on intracellular localization. We quantified the distribution of *S*. Typhimurium relative to the Golgi complex by measuring the distance between intracellular *S*. Typhimurium and the Golgi complex 8 hours after infection in HeLa cells immunostained for S. Typhimurium and Golgin-97 (Fig 3).

Consistent with previous reports, the deletion of Δ*sseFG* alters SCV localization such that Δ*sseFG* mutants are scattered throughout the host cell cytoplasm, rather than remaining in close proximity to the Golgi apparatus like wild type *S*. Typhimurium (Fig 3A, strains ii and i, respectively) [21,35,48,49,51,54,55]. The additional deletion of *steA* (resulting in the Δ*sseFG*Δ*steA* triple-effector deletion mutant, strain iii in Fig 3A) does not significantly alter *S*. Typhimurium positioning relative to the Golgi as compared to the Δ*sseFG* double deletion mutant, which is consistent with the findings of Domingues *et al*., (2014). Further deletion of PipB2 (resulting in the Δ*sseFG*Δ*steA*Δ*pipB2* quadruple-effector deletion mutant, strain iv in Fig 3) results in a strain that remains closer to the Golgi than wild type *S*. Typhimurium. Δ*pipB2* and Δ*steA* single-effector deletion mutants have previously been shown to be positioned close to the nucleus at 8–14 hours-post infection and a Δ*sseF*Δ*pipB2* double deletion mutant is found scattered throughout the cytosol [19,53]. Our results indicate potential interplay between SteA and PipB2 to promote movement away from the Golgi as both effectors must be deleted in the Δ*sseFG* background to maintain close apposition to the Golgi. Subsequent sequential deletions of *sopD2*, *sseJ*, and *sifA* (Fig 3A, strains v, vi, and vii, respectively) do not impact intracellular localization of these *S*. Typhimurium mutant strains as they all reside very close to the Golgi. This is unexpected as the single-effector deletion mutants Δ*sseFG*, Δ*sopD2*, and Δ*sifA* all have a scattered distribution SCVs throughout the host cell (Fig 3A, strains ii, xiii, and xiv, respectively). In fact, strains Δ*sseFG*Δ*steA*Δ*pipB2*Δ*sopD2*, Δ*sseFG*Δ*steA*Δ*pipB2*Δ*sopD2*Δ*sseJ*, Δ*sseFG*Δ*steA*Δ*pipB2*Δ*sopD2*Δ*sseJ*Δ*sifA* are positioned closer to the Golgi than even wild type *S*. Typhimurium. The PipB2-dependent SCV scattering was complemented when a plasmid expressing PipB2 was introduced in the Δ*sseFG*Δ*steA*Δ*pipB2* mutant strain (Fig 3B). All the remainder of the multiple-effector deletion strains in Fig 3, apart from Δ*sseFG*Δ*steA*Δ*sseJ*, have altered SCV positioning relative to wild type. Our results indicate that these effectors are involved in keeping intracellular *S*. Typhimurium close to the Golgi and that it seems likely that there is interplay between PipB2 and SteA which plays a critical role in SCV localization during infection.

### Multiple-effector deletion mutants of *S*. *Typhimurium* do not replicate in macrophages

The effectors SseJ, SopD2, SifA, PipB2, and SteA modulate SCV membrane dynamics to promote intracellular replication [32]. In macrophages, *S*. Typhimurium that escape the SCV and enter the host cell cytoplasm are eliminated by host defenses [56]. Therefore, maintaining the SCV membrane is critical to replication in macrophages. Previous studies have demonstrated that multiple T3SS2-secreted effectors, including the seven effectors in this study, contribute to replication within mouse macrophages [25,57]; specifically, the single-effector deletion mutants *sifA*, *sseJ*, *sopD2*, and *sseFG* exhibit decreased replication relative to wild type *S*. Typhimurium [57]. Given that several effectors are implicated in promoting replication in macrophages, we wanted to evaluate if our effectors of interest act independently, sequentially, or cooperatively to promote intramacrophage replication.

**Fig 4:**
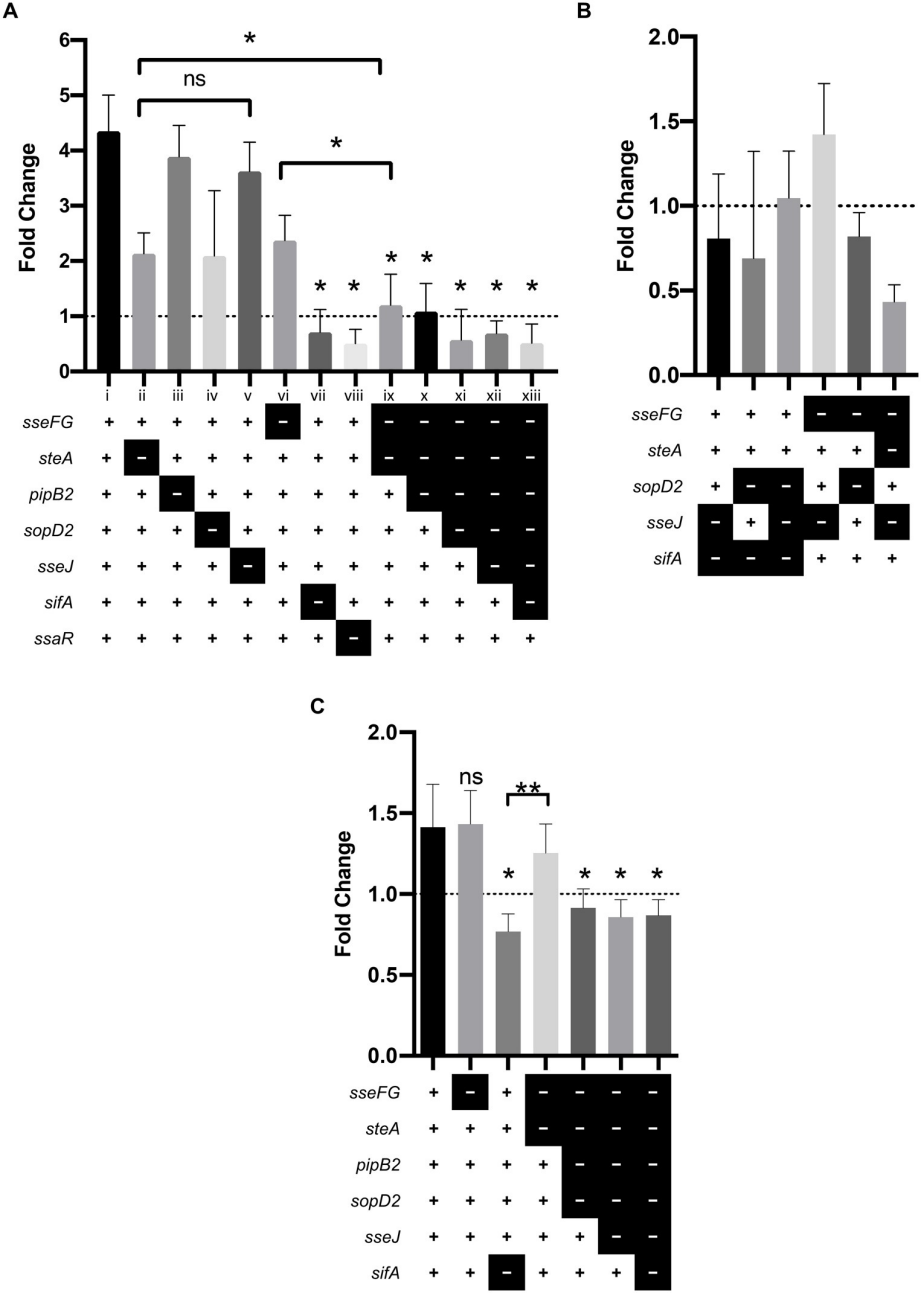
Multiple effectors are required for replication in macrophages. Strain legend: “+” = gene present, “-” = gene deleted. A “+” for all genes indicates wild type. **(A)** Replication of *S*. Typhimurium single- and sequential- deletion mutants in RAW 264.7 macrophages. RAW 264.7 cells were infected with the indicated strains at a MOI of 10. Fold change was determined by dividing CFU counts at 24 hours post-infection by CFU counts at 2 hours post-infection. The average fold change ± standard deviation for three experiments is shown (*n* = 3). An asterisk indicates a significant difference (*p* < 0.02) between the indicated mutant strain and WT or other strain if indicated by 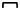 as determined by a Kruskal-Wallis one-way ANOVA with Dunn’s multiple comparison post-test. ns = not significant. **(B)** Replication of *S*. Typhimurium multiple-effector deletion mutants in RAW 264.7 macrophages. Experiment was performed and analyzed as described in (A). **(C)** Replication of *S*. Typhimurium strains in THP-1 monocytes. THP-1 monocytes were infected with select *S*. Typhimurium strains and results analyzed as described in (A). Representative results from one experiment is shown. An asterisk indicates a significant difference (*p* < 0.03) between the indicated mutant strain and WT or other strain if indicated by 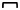 as determined by a Kruskal-Wallis one-way ANOVA with Dunn’s multiple comparison post-test. ns = not significant.

RAW 264.7 mouse macrophages were infected with our library of deletion mutants and CFUs enumerated at 2 hours and 24 hours post-infection. Most single-effector deletion mutants replicated in RAW 264.7 cells as indicated by a fold change greater than 1, while the T3SS2-secretion negative control Δ*ssaR* mutant and the Δ*sifA* mutant did not replicate which is consistent with previous reports (Fig 4A) [24,58]. Conversely, the sequential deletion mutants (strains ix-xiii in Fig 4A) were unable to replicate as were the multiple-effector deletion mutants shown in Fig 4B.

We expected that sequential and successive deletion of effectors would have a cumulatively negative impact on intramacrophage replication. As such, we expected a strain with three effectors deleted would experience decreased replication as compared to a strain with only two effectors deleted. However, while deletion of a single effector is permissive of intramacrophage replication, deletion of two or more effectors results in an inability to replicate within the macrophage. For example, the double-effector deletion strain Δ*sseFG* (considered a single-deletion mutant, Fig 4A strain vi) and the single-effector deletion strain Δ*steA* (Fig 4A, strain ii) are able to replicate within RAW 264.7 cells (fold change > 1), however the triple-effector deletion mutant Δ*sseFG*Δ*steA* (Fig 4A, strain ix) is unable to replicate (fold change < 1). We found a similar effect with select multiple-effector deletion mutants in human THP1 monocytes (Fig 4C). The inability of the sequential- and multiple-effector deletion mutants to replicate within macrophages was not driven by the specific effectors deleted, but rather by the number of effectors deleted. Simply put, intramacrophage replication does not occur when two or more of the effectors examined in this study are deleted. We can therefore conclude that intramacrophage replication is driven by the actions of multiple effectors.

### Multiple effector deletion mutants of *S*. *Typhimurium* have impaired virulence in a mouse model of infection

We have demonstrated that these seven effectors (SseF, SseG, SteA, PipB2, SopD2, SseJ, and SifA) are all required to establish an intracellular replicative niche within both epithelial cells and macrophages. While these models provide insight into the cellular events within host cells, they do not provide any information regarding virulence. We therefore investigated the contribution of these seven effectors to virulence in an *in vivo* infection model. We chose to use a low-dose streptomycin pre-treatment murine model of gastroenteritis as it more closely models *S*. Typhimurium infections in humans and produces consistent results in our hands. In this model, pre-treatment of C57BL/6 mice with low-dose of streptomycin induces susceptibility to gastroenteritis upon infection with wild type *S*. Typhimurium [59].

**Fig 5:**
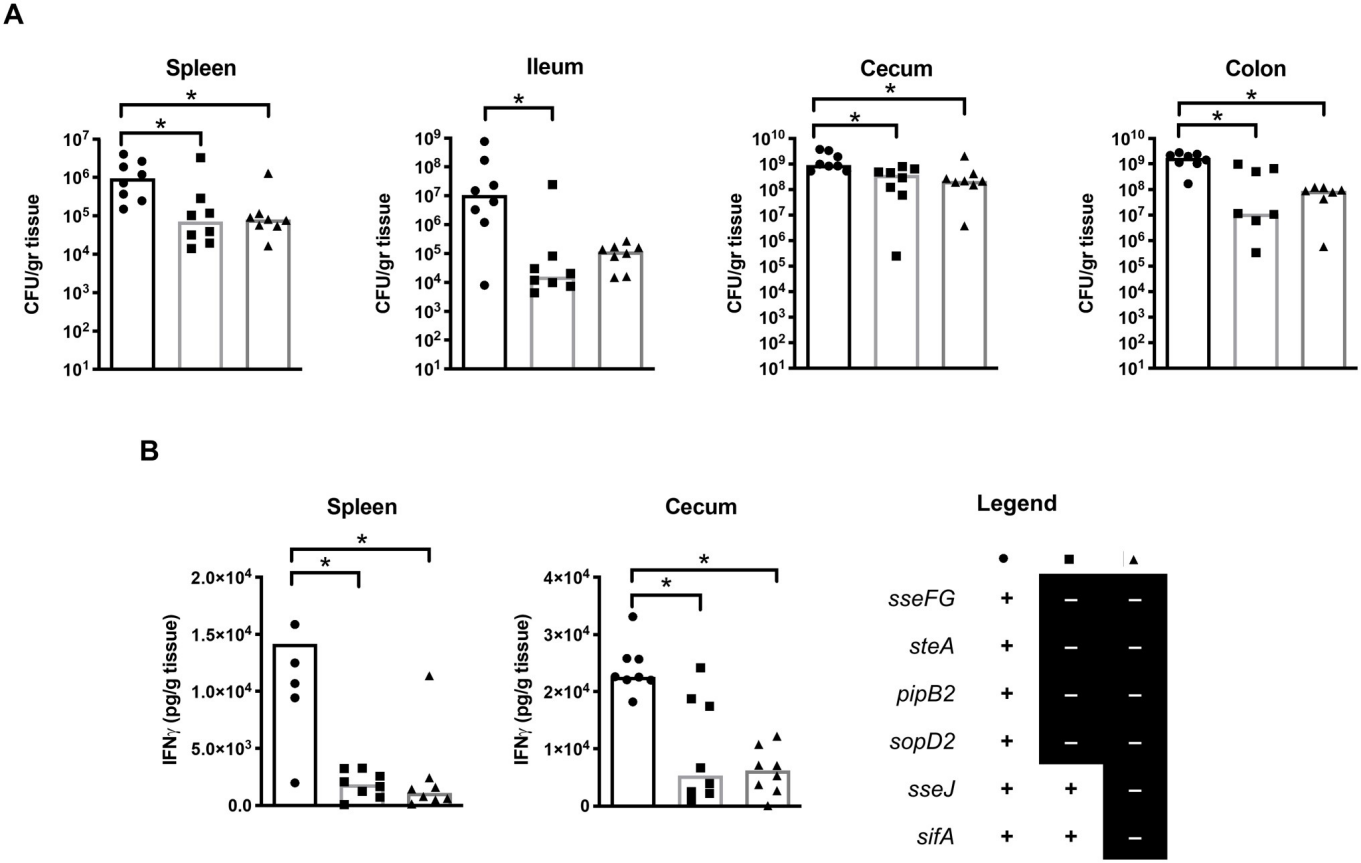
Gastroenteritis model of S. Typhimurium infection. Mice were treated with streptomycin for two days prior to oral infection with select *S*. Typhimurium strains to induce gastroenteritis as per Sekirov et al., 2008. Strain legend found on bottom right of figure: “+” = gene present, “-” = gene deleted. A “+” for all genes indicates wild type. A single asterisk indicates a significant difference between the indicated strains (*p* < 0.03) as determined by a Kruskal-Wallis one-way ANOVA with Dunn’s multiple comparison post-test. **(A)** Bacterial counts were recovered from systemic and intestinal organs of mice three days post-infection. Counts given represent colony forming units per gram of tissue. The median CFU/g for three separate experiments is shown with individual data points visible to accurately represent the spread of the data. **(B)** IFNγ-levels as determined by ELISA. The median amount of IFNγ (pg of IFNγ/g of tissue) is shown with individual data points visible to accurately represent the spread of the data.

Streptomycin-treated mice were infected with either wild type SL1344, Δ*sseFG*Δ*steA*Δ*pipB2*Δ*sopD2*, or Δ*sseFG*Δ*steA*Δ*pipB2*Δ*sopD2*Δ*sseJ*Δ*sifA* multiple-effector deletion strains. These two multiple-effector deletion strains were selected as they both exhibit reduced frequency of LAMP1^+^-tubulation and an inability to replicate in macrophages. *S*. Typhimurium colonization in intestinal and systemic sites were determined at three days post-infection by CFU counts from the spleen, cecum, ileum, and colon. Both multiple-effector deletion strains colonized the spleen, cecum, and colon significantly less than the wild type strain (Fig 5A). Both multiple-effector deletion strains also colonized the ileum to a lesser extent than the wild type strain, though the Δ*sseFG*Δ*steA*Δ*pipB2*Δ*sopD2*Δ*sseJ*Δ*sifA* was not statistically significantly different from wild type (Fig 5A). There was no significant difference in colonization at the four sites between Δ*sseFG*Δ*steA*Δ*pipB2*Δ*sopD2* and Δ*sseFG*Δ*steA*Δ*pipB2*Δ*sopD2*Δ*sseJ*Δ*sifA*. This suggests that successful colonization, at both intestinal and systemic sites, requires the action of multiple effectors.

One of the hallmarks of non-typhoidal salmonellosis infection is acute intestinal inflammation [60]. Therefore, elevated levels of gastrointestinal inflammation—reflected by the levels of IFNγ—indicate *S*. Typhimurium infection within the intestinal epithelium. We found significantly decreased IFNγ in the spleen and cecum of mice infected with the multiple-effector deletion strains Δ*sseFG*Δ*steA*Δ*pipB2*Δ*sopD2* and Δ*sseFG*Δ*steA*Δ*pipB2*Δ*sopD2*Δ*sseJ*Δ*sifA* as compared to mice infected with wild type strain (Fig 5B). The additional deletion of *sifA* and *sseJ* to the Δ*sseFG*Δ*steA*Δ*pipB2*Δ*sopD2* strain does not further decrease colonization or inflammation, mirroring our results in the macrophage infection models (both RAW 264.7 and THP-1 cells). This suggests that the multiple-effector deletion strains did not elicit as strong of an inflammatory response as the wild type strain. The combination of decreased colonization and decreased inflammation induced by the multiple-effector deletion strains suggests that multiple effectors are required for both colonization and virulence of *Salmonella* within a gastroenteritis model of infection.

## Discussion

The importance of T3SS2-secreted effectors during infection is widely recognized, but the precise biochemical activity and function of many of these effectors is poorly understood. Previous attempts to identify the functions and targets of T3SS2-secreted effectors often involve studying effectors separately [22,55,61,62]; however increasing evidence suggests that the effectors have overlapping yet distinct roles during infections. As infections involve dynamic and complex processes, the effect of one effector may require prior action by another effector, or their activities may be linked. It is therefore necessary to study effector activities in the presence or absence of other related effectors to discover the precise function of each effector.

SIF biogenesis is a complex and dynamic process involving the action of several effectors as shown by multiple studies [16,43,45]. Here, we demonstrate the complexity of LAMP1^+^-tubule extension (*i*.*e*. SIF biogenesis) by infecting HeLa cells with a library of single-effector and multiple-effector deletion strains. Most single-effector deletion mutant strains are capable of forming LAMP1^+^-tubules at a frequency of at least 25% relative to wild type, whereas all 11 multiple-effector deletion strains fail to induce LAMP1^+^-tubules at a frequency greater than 10% relative to wild type. The fact that single-effector deletion mutants form more LAMP1^+^-tubules as compared to the multiple-effector deletion mutants implies that more than one effector is required to extend LAMP1^+^-tubules and that at least two or more of the effectors are working in conjunction with one another. Furthermore, the sequential deletion of effectors does not decrease the frequency of LAMP1^+^-tubule formation in a step wise manner indicating unequal contribution by each effector to this process. The severe and non-cumulative defects in LAMP1^+^-tubule formation observed in the multiple-effector deletion mutants strongly suggests that extensive LAMP1^+^-tubule extension requires multiple effectors mediating the process.

Previous studies show that multiple T3SS2-secreted effectors are required to mediate LAMP1^+^-tubule extension [31,35]. While one study indicates that ectopically expressed *sifA* in HeLa cells is sufficient to induce LAMP1^+^-tubulation, others studies report significantly higher frequencies of LAMP1^+^-tubulation when *sifA* is co-expressed with either *sopD2* or *sseJ* [27,31]. SifA and SseJ cooperate through interactions with the host kinesin-binding protein SKIP and RhoA family GTPases to induce LAMP1^+^-tubulation [31]. We observed similar frequencies of LAMP1^+^-tubulation in a strain with functional SifA and SseJ (Δ*sseFG*Δ*steA*Δ*pipB2*Δ*sopD2*) and a strain with only functional SifA (Δ*sseFG*Δ*steA*Δ*pipB2*Δ*sopD2*Δ*sseJ*), while LAMP1^+^-tubules were not observed in the strain that lacks all seven effectors of interest (Δ*sseFG*Δ*steA*Δ*pipB2*Δ*sopD2*Δ*sseJ*Δ*sifA*). From this we can conclude that SifA is sufficient to induce LAMP1^+^-tubule formation on its own. However, the frequency of LAMP1^+^-tubulation was not significantly higher when both SifA and SseJ are functional than with SifA alone, suggesting that the previously described cooperative actions between SifA and SseJ, resulting in the increased LAMP1^+^-tubulation, possibly involves other *S*. Typhimurium effectors or other host proteins to increase the frequency of LAMP1^+^-tubules. The discrepancy between our results and others, regarding the cooperation between SifA and SseJ, may be explained by previous studies observing increased LAMP1^+^-tubule frequencies with ectopically expressed SifA and SseJ [30,31] whereas our study is in the context of a *S*. Typhimurium infection. Our results strongly suggest that multiple T3SS2-secreted effectors are required to facilitate efficient formation and extension of LAMP1^+^-tubules.

Previous studies have demonstrated that neither the Δ*sifA* single-effector deletion mutant nor the Δ*sifA*Δ*sseJ* double-effector deletion mutant form SIFs. However, the Δ*sifA* mutant escapes the SCV while the Δ*sifA*Δ*sseJ* mutant remains in the SCV. [15,24,30,63,64]. While the N-terminal domain of SifA is required for interactions with the host protein SKIP (PLEKHM2) and PLEKHM1 [48,62,65–67], the C-terminal domain promotes LAMP1 recruitment to *Salmonella*-induced tubules via interactions with the GTPase Arl8b [68]. Studies have demonstrated that Arl8b controls membrane fusion events with late endocytic compartments and is associated with LAMP1 accumulation in SIFs [68,69]. In our study, the Δ*sifA* deletion mutant was unable to extend LAMP1^+^-tubules, yet we observed LAMP1^+^-tubules in HeLa cells infected with Δ*sifA*Δ*sseJ* and Δ*sifA*Δ*sopD2* strains, suggesting that LAMP1 recruitment, and subsequent LAMP1^+^-tubule extension, can occur via a SifA-independent mechanism. Further work is required to determine if LAMP1 recruitment in these strains is mediated by an additional effector interacting with Arl8b, or if an alternative Arl8b-independent mechanism is at play. The fact that the additional deletion of *sseJ* or *sopD2* in the Δ*sifA* background restores the ability to extend LAMP1^+^-tubules indicates potential antagonistic action between SseJ or SopD2 and other effectors that mediate SIF biogenesis.

The low frequency of LAMP1^+^-tubule extension observed in multiple-effector deletion mutants lacking Δ*sseFG* may be directly related to the role of SseF and SseG during infection. Both SseF and SseG, while not required for the formation of single membrane SIFs, are required for the conversion of single-membraned SIFs (also known as pseudo-SIFs) to double-membrane SIFs [43]. The inability to convert from single- to double-membraned SIFs may explain the thinner appearance of SIFs in cells infected Δ*sseF/G* strains as compared to wild type infected cells [36,46]. The methods used in our study may fail to detect these thinner pseudo-SIFs, and may therefore account for low frequency of LAMP1^+^-tubules in the Δ*sseFG* mutant strains.

Multiple studies have established that precise intracellular SCV positioning plays a key role during infection [48,49,54,70,71]. Mutant *S*. Typhimurium strains that fail to cluster near the Golgi at 8 hours-post infection in epithelial cells have lower frequencies of LAMP1^+^-tubule extension and impaired intracellular replication [35,49,72]. The *sseF* and/or *sseG* deletion mutants are found scattered throughout the host cell’s cytoplasm which could be a consequence of dysregulated microtubule motors [21]. Alternatively, the scattered phenotype caused by *sseF/G* deletion could also result from the absence of SseF and SseG mediated tethering to the Golgi-associated protein ACBD3 [55]. SifA and PipB2 also play a role in SCV localization during infection. T3SS2-secreted effector SifA inserts into the SCV membrane where it binds to the C-terminal PH domain of SKIP (PLEKHM2) [48,73]. Meanwhile, PipB2 tethers auto-inhibited kinesin-1 to the SCV membrane [22] in a process involving the small GTPase Arl8b [69,74]. Kinesin-1 is then activated by binding to the SifA-SKIP complex [65]. Deletion of *pipB2* prevents centrifugal displacement of SCVs at later timepoints in infection [19]. SteA may play a role in SCV positioning as it is thought to activate kinesin-1 or inhibit dynein [53]. While we have many pieces of the puzzle, the exact mechanisms controlling SCV localization during infection remains unclear.

A previous study investigated the role of up to three effectors on the intracellular localization of *S*. Typhimurium during infection. The authors found that intracellular wild type, Δ*steA*, and Δ*pipB2* single-effector deletion mutants tend to reside close to the Golgi-apparatus at up to 14 hours post-infection, whereas any multiple deletion mutant that also had *sseF* and/or *sseG* deleted were more likely to be scattered throughout the host cell cytosol [19,53]. We found that the additional deletion of *pipB2* in the Δ*sseFG*Δ*steA* background (resulting in Δ*sseFG*Δ*steA *Δ*pipB2*) restores SCV localization close to the Golgi-apparatus. Subsequent deletion of additional effectors in the Δ*sseFG*Δ*steA*Δ*pipB2* background did not alter this close apposition of the SCV to the Golgi. Conversely, SCVs in mutant strains with functional PipB2 and SteA tend to be scattered throughout the host cell cytosol. These results suggest that PipB2 may be the first effector in a series of events involving SteA that leads to outwards centrifugal movement of SCV at 8 hours post-infection (and later time points as well) and without the initial action of PipB2, the SCV remains in close proximity to the Golgi. The intracellular positioning of *Salmonella* must therefore rely on a delicate balance of effector actions to precisely regulate SCV positioning during infection. We can therefore conclude that multiple effectors are required to regulate positioning of the SCV during infection.

The question remains as to what the link is, if any, between SCV localization, LAMP1^+^-tubule extension, and intracellular replication? Does effector deletion alter SCV localization, which directly impairs LAMP1^+^-tubule extension, thereby limiting intracellular replication? Or does effector deletion itself impair LAMP1^+^-tubule extension, resulting in decreased intracellular replication and altered SCV localization is merely a coincidental phenotype? An example that brings causality into question is the effect of deletion of *sseF* and *sseG* (Δ*sseFG*). We, and others, have shown that deletion of *sseFG* results in altered SCV localization and decreased LAMP1^+^-tubule formation in HeLa cells, as well as reduced intracellular replication. Other studies have found that Δ*sseFG* strains also have altered SIF morphology [36,43]. SIFs (LAMP1^+^-tubules) are necessary for supplying intravacuolar *S*. Typhimurium with nutrients [20]. Intravacuolar *S*. Typhimurium forms SIFs by converting the host’s endosomal system into SIFs to siphon nutrients from the host [18]. So then, does the replication defect of the Δ*sseFG* double mutant result from altered SCV localization, or does the altered SIF morphology itself limit nutrient acquisition from the host causing impaired intracellular replication? Alternatively, does altered SIF morphology result directly from altered SCV localization, impacting interactions with the host’s endosomal system, and thus limiting nutrient acquisition, resulting in impaired intravacuolar replication? Further studies are necessary to elucidate the cause and effect of these processes. Intracellular localization, SIF formation, and replication are likely very intertwined and deletion of one effector critical to one of these processes could dramatically impact the others.

Multiple effectors are required to promote intracellular replication in macrophages. Most single-effector deletion mutants replicate within both RAW 264.7 macrophages and THP-1 monocytes, whereas multiple-effector deletion mutants do not. The precise reason for impaired replication is unclear, however a potential explanation is that deletion of multiple effectors alters interactions with the host’s endocytic pathway which would then alter or limit SIF formation and thereby decrease nutrient acquisition from the host [18,20,43,75]. The fact that all multiple-effector deletion mutants have impaired intramacrophage replication regardless of the effectors deleted, suggests that all effectors are required in combination with each other to successfully replicate within host cells.

The importance of these SPI-2 effectors during infection was reinforced by examining colonization and inflammatory response in an *in vivo* infection model. It has been reported that SifA is not required to induce inflammation in the colon of mice [76]; however another group showed that strains lacking SifA in addition to other T3SS2-decreted effectors (SseF, SseJ, SteA, and SpvB) have dramatically reduced inflammation during infection suggesting that intestinal inflammation requires the cooperative effects of at least these five effectors [77]. In line with these findings, both multiple-effector deletion strains in our study Δ*sseFG*Δ*steA*Δ*pipB2*Δ*sopD2* and Δ*sseFG*Δ*steA*Δ*pipB2*Δ*sopD2*Δ*sseJ*Δ*sifA* exhibited decreased virulence in a low-dose streptomycin pre-treatment mouse model of gastroenteritis. Both multiple-effector deletion strains are impaired to a similar degree with respect to colonization and inflammation in mice as compared to the mice infected with wild type *S*. Typhimurium. This means that SifA and SseJ are insufficient to mount a successful infection without the other five effectors of interest: SseF, SseG, PipB2, SteA, and SopD2. The impaired colonization observed in the two multiple-effector deletion mutants used in the mouse model of infection means that deletion of these effectors either impacts the ability of these strains to invade the host cells, the ability to replicate within host cells, or the ability to evade the host’s immune system. The decreased inflammation in mice infected with either multiple-effector deletion strain also suggests that these strains may not invade host cells as efficiently as wild type strains, or they were easily eliminated from the host before the end point of the experiment. Similar to the conclusions of Matsuda *et al*. (2019), we can surmise that the seven effectors of interest in this study are required to mount a successful infection in a mouse model of gastroenteritis.

We have shown that the processes of SIF biogenesis, intracellular localization, replication in macrophages, and colonization and inflammation in a mouse model, all require multiple effectors present and working together to successfully mount an infection. Our study highlights the fact that not one single effector of our seven of interest, is solely responsible for mediating complex infection phenotypes. It seems likely that several effectors act on the same process, either in conjunction with one another, or in a sequential manner. If these effectors work sequentially, then deletion of an effector that works early within the pathway will have dramatic results. As an example: deletion of PipB2 helps *S*. Typhimurium mutants remain very close to the Golgi during infection regardless of the presence or absence of other effectors, indicating that PipB2 likely acts early within the pathway. Similarly, these effectors could interact within much larger complexes, and deletion of a key effector could render the entire complex ineffective. If we want to elucidate the exact mechanisms underpinning *Salmonella*’s intracellular replicative niche, we must study the role of each effector in the context of other effectors, rather than deleting single effectors, or transfecting a single effector into tissue culture cells and examining their effect. Further studies are required to examine how these effectors interact with each other, or on similar host processes.

## Materials and methods

### Ethics statement

All animal experiments were performed according to protocol number A13-0265 approved by the University of British Columbia’s Animal Care Committee and in direct accordance with the Canadian Council of Animal Care (CACC) guidelines. Mice were euthanized at 3 days post-infection.

### Bacterial strains and culture conditions

Bacterial strains used in this work are described in Table 1. All strains were routinely grown in Luria-Bertani (LB) medium at 37°C with shaking. For growth of the *E*. *coli* MFD*pir* strain, media was supplemented with DL-2,6-Diaminopimelic acid (DAP) at a final concentration of 0.3 mM when appropriate. Antibiotics were used at the following concentration when required: streptomycin 50 *μ*g/mL, chloramphenicol 30 *μ*g/mL.

### Plasmid construction

Plasmids constructed and used in the study are listed in Table 2; primers used are described in Table 3. All plasmids were constructed using the Gibson Assembly method of cloning [78]. Complementation vectors and gene deletion vectors were routinely maintained in *E*. *coli* DH10B and MC1061 *λpir* respectively.

**Table 2:** Plasmids used in this study.

Plasmid Designation	Relevant Characteristics/Genotype	Source/Reference
pRE112	*cat sacB oriV*_*RGKγ*_*oriT*_*RP4*_ Cm^R^	[33]
pACYC184	*ori*P15A, Tet^R^, Cm^R^	[79]
pRE112-ΔsseFΔsseG	Upstream region of *sseF* and downstream region of sseG from *S*. Typhimurium SL1344 in pRE112	This study
pRE112-ΔsteA	Upstream and downstream regions of *steA* region from *S*. Typhimurium in pRE112	This study
pRE112-ΔpipB2	Upstream and downstream regions of *pipB2* region from *S*. Typhimurium in pRE112	This study
pRE112-ΔsopD2	Upstream and downstream regions of *sopD2* region from *S*. Typhimurium in pRE112	This study
pRE112-ΔsseJ	Upstream and downstream regions of *sseJ* region from *S*. Typhimurium in pRE112	This study
pPIPB2	*pipB2* under the control of its native promoter in pACYC184	This study

Cm^R^ = Chloramphenicol resistance, Tet^R^ = Tetracycline resistance

**Table 3:** Primers used in this study.

	Forward oligonucleotide	Reverse Oligonucleotide
*sseF* 5’ flanking	GAACTGCATGAATTCCCGGGCTGGACAGTTTTATCCGCCG	CGGTATATACCTGAAAACGATTACATATTTCGTTCTGTTATTTAAGCAATAAG
*sseG 3’* flanking	GAAATATGTAATCGTTTTCAGGTATATACCGG	CAAGCTTCTTCTAGAGGTACCGAAATAACAGACGCAGCGCC
*steA 5’* flanking	GAACTGCATGAATTCCCGGGCCATCGCTTTGTGATACCCC	CATATCCTACTCCTTCAAATTTTGCTC
*steA 3’* flanking	CAAAATTTGAAGGAGTAGGATATGTAAAAAGCGTTTATGTTTAGCC	CAAGCTTCTTCTAGAGGTACCCGGGATGAGACAGAATGACC
*pipB2 5’* flanking	GAACTGCATGAATTCCCGGGGCTGCATCGTCATACTACGG	CATATATTTTCTCCCAGAGACAGCAAC
*pipB2 3’* flanking	GTCTCTGGGAGAAAATATATGTAGCCCTTTTTGACGTAAATCTG	CCAAGCTTCTTCTAGAGGTACCCCTGGTAATATTTATCAGGCG
*sopD2 5’* flanking	GTGAACTGCATGAATTCCCGGGGGGGTTTATGGACACATTCC	CTTTTTACATAATAACTCCCTTGATTATTTACCG
*sopD2 3’* flanking	GTAAATAATCAAGGGAGTTATTATGTAAAAAGTCATTAAAAAGGCC	CAAGCTTCTTCTAGAGGTACCGTTCTGACCATTACTTCTAACG
*sseJ 5’* flanking	CAAGCTTCTTCTAGAGGTACCCCCACTCCCCACGCTATTATG	CATAGTGTCCTCCTTACTTTATTAAACACG
*sseJ 3’* flanking	CGTGTTTAATAAAGTAAGGAGGACACTATGTAAAGTTCCATCGGCTGCGG	ATGAATTCCCGGGAGAGCTCCCTGGCAACGGTTAAGGTGG
*sifA 5’* flanking	CAAGCTTCTTCTAGAGGTACCCACCCCGAGCGCCGTTATTATC	CGTCTGATTTTACATATTAATCTCACTTATACTGGAG
*sifA 3’* flanking	GAGATTAATATGTAAAATCAGACGACGCTTTCTCAGACG	ATGAATTCCCGGGAGAGCTCGACCGTGACGACCACAAACG
pRE112 plasmid backbone	GGTACCTCTAGAAGAAGCTTGGGA	CCCGGGAATTCATGCAGTTCAC
pACYC184 plasmid backbone	GCGGCCGCTCGATACCCATACG	CCCGAGATGCGCCGCGTGC
*pipB2* complementation	GCACGCGGCGCATCTCGGGGAGTTGCAGGAAGGCGGCAAGC	GTATGGGTATCGAGCGGCCGCAATATTTTCACTATAAAATTCGTTAAAGAGTG

The pRE112 plasmid backbone used for all gene deletion constructs was produced using the pRE112 backbone primer set to amplify linear pRE112 from the KpnI to SacI unique restriction sites (final size of 5749 bp). pRE112 plasmid backbone was subsequently digested with DpnI (NEB) to remove any remaining circular template DNA. To generate complete, unmarked deletions, the upstream homologous region of target genes up to an including the start codon, and the downstream homologous region of the target gene starting with the stop codon were amplified by PCR from the chromosomal DNA of wild type SL1344.

As *sseF* and *sseG* are in an operon [34], plasmid pRE112-ΔsseFΔsseG was generated by amplifying the upstream region of *sseF* and the downstream region of *sseG* using primer pairs *sseF* 5’ flanking and *sseG* 3’ flanking respectively. PCR products were ligated into the pRE112 vector using Gibson Assembly. Plasmids pRE112-ΔsteA, pRE112-ΔpipB2, pRE112-ΔsopD2, pRE112-ΔsseJ, and pRE112-ΔsifA using the respective 5’ flanking and 3’ flanking primers pairs shown in Table 3.

### Generation of mutants by allelic exchange

Unmarked complete deletion mutants were generated as previously described [25]. Briefly, MFD*pir* strain transformed with the gene deletion plasmids were conjugated with different SL1344 based-strains. The unmarked SL1344 gene deletion were constructed by inserting the pRE112 plasmid constructs into the SL1344 chromosome. Post-conjugation single crossover mutants between pRE112 constructs and the SL1344 chromosome were selected on LB agar plates containing chloramphenicol. Sucrose counter-selection was performed as previously described [33] to select for the second crossover event, thus effectively deleting the gene of choice, leaving only the start and stop codons.

The unmarked SL1344 mutant strain Δ*sseFG* was constructed by inserting the homologous regions from the pRE112-ΔsseFΔsseG plasmid into the wild type chromosome. The unmarked SL1344 mutant strain Δ*sseFG*Δ*steA* was constructed by inserting the homologous regions from the pRE112-ΔsteA into the Δ*sseFG* mutant chromosome. The unmarked SL1344 mutant strain Δ*sseFG*Δ*steA*Δ*pipB2* was constructed by inserting the homologous regions from the pRE112-ΔpipB2 into the Δ*sseFG*Δ*steA* mutant chromosome. The unmarked SL1344 mutant strain Δ*sseFG*Δ*steA*Δ*pipB2*Δ*sopD2* was constructed by inserting the homologous regions from the pRE112-ΔsopD2 into the Δ*sseFG*Δ*steA*Δ*pipB2* mutant chromosome. The unmarked SL1344 mutant strain Δ*sseFG*Δ*steA*Δ*pipB2*Δ*sopD2*Δ*sseJ* was constructed by inserting the homologous regions from the pRE112-ΔsseJ into the Δ*sseFG*Δ*steA*Δ*pipB2*Δ*sopD2* mutant chromosome. The unmarked SL1344 mutant strain Δ*sseFG*Δ*steA*Δ*pipB2*Δ*sopD2*Δ*sseJ*Δ*sifA* was constructed by inserting the homologous regions from the pRE112-ΔsifA into the Δ*sseFG*Δ*steA*Δ*pipB2*Δ*sopD2*Δ*sseJ* mutant chromosome. Homologous regions from plasmid pRE112-ΔsseJ was introduced into the chromosomes of SL1344 mutant strain Δ*sseFG* and Δ*sifA*, creating strains Δ*sseFG*Δ*sseJ* and Δ*sifA*Δ*sseJ* respectively. Homologous regions from the plasmid pRE112-ΔsopD2 was introduced into the chromosomes of SL1344 mutant strains Δ*sseFG* and Δ*sifA*, creating strains Δ*sseFG*Δ*sopD2* and Δ*sifA*Δ*sopD2* respectively. The strains Δ*sifA*Δ*sseJ*Δ*steA* and Δ*sifA*Δ*sseJ*Δ*sopD2* were generated by incorporating the homologous regions of the plasmids pRE112-ΔsteA and pRE112-ΔsopD2, respectively into the Δ*sifA*Δ*sseJ* mutant chromosome. Successful gene deletions were verified by PCR and DNA sequencing.

### Cell lines

HeLa (ATCC^®^ CCL-2^™^), RAW 264.7 (ATCC^®^ TIB-71^™^), and THP-1 (ATCC^®^ TIB-202™) cells were directly obtained from ATCC. All cell lines were routinely maintained at 37°C in a 5% CO_2_ atmosphere. HeLa and RAW 264.7 cells were cultured in Dulbecco’s Modified Essential Medium (DMEM) (Hyclone) containing 10% (v/v) heat-inactivated fetal bovine serum (FBS) (Gibco), 1% (v/v) Glutamax (Gibco), and 1% (v/v) nonessential amino acids (Gibco). HeLa and RAW 264.7 cells were used up to passage 15. THP-1 cells were routinely maintained at a density of 2 x 10^5^ to 1 x 10^6^ cells/mL in Roswell Park Memorial Institute (RPMI) 1640 Medium (Gibco) supplemented with 10% (v/v) heat inactivated FBS, and 1% (v/v) nonessential amino acids. THP-1 cells were used up to passage 10.

### HeLa cell infections

HeLa cells were seeded on 12 mm diameter glass coverslips in 24-well plates (Corning) at a density of 5 x 10^4^ cells/well, 16–24 hours prior to infection. Overnight bacterial cultures were diluted 1:33 in LB without antibiotic and incubated for 3 hours at 37°C with shaking (late log-phase cultures). 1 mL of bacterial cultures were pelleted and resuspended in Dulbecco’s Phosphate-Buffered Saline (DPBS) (Hyclone), subsequently diluted in DMEM and added to the HeLa cells at a multiplicity of infection (MOI) of ≈ 100:1. The infection was allowed to proceed for 15 minutes at 37°C in 5% CO_2_. Non-internalized bacteria were removed by three washes in DPBS and cells incubated in growth media containing 100 *μ*g/mL gentamicin until 2 hours post-infection, followed by growth media containing 10 *μ*g/mL gentamicin for the remainder of the experiment. HeLa cells were infected for a total of 8 hours.

### RAW 264.7 cell infections

RAW 264.7 cells were seeded in 24-well plates at a density of 1 x 10^5^ cells/well 16–24 hours prior to infection. Overnight bacterial cultures (stationary phase) were pelleted and resuspended in DPBS, and subsequently opsonized in DPBS containing 10% normal mouse serum for 20 min at 37°C. Opsonized bacteria were diluted in DMEM and added to the monolayers at a MOI of ≈ 10:1, centrifuged at 170 *g* for 5 min at room temperature and incubated for 25 min at 37°C in 5% CO_2_. Non-internalized bacteria were removed by three washes in DPBS and cells incubated in growth media containing 100 *μ*g/mL gentamicin until 2 hours post-infection, followed by growth media containing 10 *μ*g/mL gentamicin for the remainder of the experiment. For enumeration of intravacuolar bacteria, macrophages were lysed in lysis buffer (1% Triton X-100, 0.1% SDS (Sigma) in DPBS) for 10 minutes and serial dilutions plated on LB agar containing 50 *μ*g/mL streptomycin. CFU counts were taken at 2 hours, and 24 hours post-infection.

### THP-1 cell infections

THP-1 cells were seeded in 24-well plates at a density of 2 x 10^5^ cells/well in complete RPMI media supplemented with 100 nM phorbol myristate acetate (PMA) 16–24 hours prior to infection for differentiation. Overnight bacterial cultures (stationary phase) were pelleted and resuspended in DPBS, and subsequently diluted in RPMI and added to the THP-1 cells at a MOI of ≈ 50:1. Plates were centrifuged at 170 *g* for 5 min at room temperature and incubated for 25 min at 37°C in 5% CO_2_. Non-internalized bacteria were removed by three washes in DPBS and cells incubated in RPMI containing 100 *μ*g/mL gentamicin until 2 hours post-infection, followed by RPMI containing 10 *μ*g/mL gentamicin for the remainder of the experiment. CFU counts were taken at 2 hours, and 24 hours post-infection.

### Antibodies

The goat polyclonal anti-*Salmonella* antibody CSA-1 (Kirkegaard and Perry Laboratories) was used at a dilution of 1:300; the mouse anti-LAMP1 antibody H4A3c developed by J.T. August and J. E. K. Hildreth, obtained from the Developmental Studies Hybridoma Bank developed under the auspices of the NICHD and maintained by the University of Iowa (Department of Biological Sciences, Iowa, USA) was used at a dilution of 1:100. The mouse monoclonal anti-Golgin 97 antibody CDF4 (Molecular Probes) was used at a dilution of 1:100. Secondary antibodies were obtained from Thermo Fisher Scientific and used at a dilution of 1:500: Alexa 488-conjugated donkey anti-mouse, and Alexa 568-conjugated donkey anti-goat.

### Immunofluorescence microscopy

Cell monolayers seeded on glass coverslips were fixed with 4% (vol/vol) paraformaldehyde in DPBS at room temperature for 10 minutes, and washed three times in DPBS. Excess paraformaldehyde was quenched in 50 mM ammonium chloride for 10 minutes at room temperature followed by two washes in DPBS.

For LAMP1^+^-tubules: Cells on coverslips were permeabilized in ice-cold acetone for 5 minutes at -20°C and then blocked in 1% Bovine Serum Albumin (BSA, wt/vol) (Sigma) in DPBS for 30 minutes at room temperature. Cells on coverslips were then incubated with primary antibodies diluted in 1% BSA in DPBS at room temperature for 1 hour followed by three washes in DPBS. Secondary antibodies diluted in 1% BSA in DPBS were added to the coverslips and incubated at room temperature for 1 hour and then washed once with DPBS. Cells were then incubated for 10 minutes at room temperature with DAPI (Invitrogen) in DPBS, followed by two DPBS washes. Cells were then washed in deionized water prior to mounting with ProLong Gold Antifade Mountant (Life Technologies) on glass slides. Microscopy was performed using Zeiss Axio Imager M2 (100x objective) and processed using Zeiss Zen Pro and ImageJ (NIH) softwares.

For Golgi-staining: Cells on coverslips were simultaneously permeabilized and blocked in 10% Normal Goat Serum (NGS) (Invitrogen) and 0.1% Triton X-100 in DPBS for 30 minutes at room temperature. Cells on coverslips were then incubated with primary antibodies diluted in 10% NGS and 0.1% Triton X-100 in DPBS and incubated at room temperature for 1 hour followed by three washes in DPBS. Secondary antibodies diluted in 10% NGS and 0.1% Triton X-100 in DPBS were then added to the coverslips and incubated at room temperature for 1 hour followed by three washes in DPBS. Coverslips were mounted on glass slides using ProLong^®^ Gold Antifade Mountant with DAPI (Life Technologies) and incubated at room temperature for 24 h prior to sealing. Microscopy was performed using Olympus IX81 microscope (100x objective) and SlideBook 4.1.0 software. Distances were quantified using ImageJ software (NIH).

### Scoring of phenotypes by microscopy

To quantify the number of infected cells with LAMP1^+^-tubules, we surveyed cells infected with *Salmonella* and immunolabelled for *Salmonella* and LAMP1. Uninfected cells were discarded from consideration. Infected cells were then scored for presence or absence of LAMP1^+^-tubules radiating outwards from a labelled *Salmonella*. The number of tubules/*Salmonella* was not considered as we were only concerned with the presence or absence of LAMP1^+^-tubules per each bacterium. At least 100 infected cells were scored blind in each experiment, and each experiment was repeated at least three times.

To quantify the distance from the Golgi, we surveyed cells infected with *Salmonella* and immunolabelled for *Salmonella* and Golgin-97. Distanced from the Golgi was enumerated by measuring the distance from the center of individual *Salmonella* cells to the center of the Golgi (*μ*M). At least 200 *Salmonella*-to-Golgi distances were measured blind in each experiment, and all experiments were repeated at least three times.

### Murine gastroenteritis model

Specific pathogen free C57BL/6 female 6-week old mice were obtained from The Jackson Laboratory (Bar harbor, Maine, USA) and housed in the animal facility at the University of British Columbia. Mice were pre-treated with 450 mg/L of streptomycin in drinking water as previously described [59]. Mice were orally gavaged with 2.8 x 10^7^ CFU/mouse from overnight cultures of wild type and mutant SL1344 strains suspended in 0.1 mL DPBS. Mice were euthanized three days post-infection by anesthesia with isoflurane followed by CO_2_ asphyxiation and tissues were aseptically harvested for further evaluation. Ceca, colons, ilea and spleens were collected in 1 mL of sterile DPBS and homogenized by a FastPrep Homogenizer (MP Biochemicals). CFU from each organ was enumerated by serial dilutions on LB agar plates containing 100 *μ*g/mL of streptomycin.

### ELISAs

Cecum and spleen homogenates were centrifuged twice for 10 minutes at 13,000 *g*, and the supernatants were collected, diluted 1:2 in DPBS, and stored at -20°C. Levels of interferon-γ (IFN-γ) were determined by enzyme-linked immunosorbent assays (ELISAs) using BD OptEIA Mouse IFN-γ ELISA set (BD Biosciences) according for the manufacturer’s instructions. IFN-γ levels were normalized to the weight of the organs.

### Statistical analysis

Statistical analysis was performed using Prism8 (GraphPad). *In vitro* infections, *S*. Typhimurium colonization in mice, and cytokine levels were analyzed. All data was found to not adhere to a Gaussian distribution using the following normality tests: Shapiro-Wilk test, and the Kolmogorov-Smirnov normality test with Dallal-Wilkinson-Lilliefor P value test. Data sets were analyzed using Kruskal-Wallis one-way analysis of variance (ANOVA) with Dunn’s multiple comparison post-test.

## Supporting information

S1 Fig Effector deletion mutants grow normally in LB. Single- and multiple-effector deletion mutants do not have impaired growth in LB liquid culture. 3 mL cultures of each strains were grown for 16–20 hours in Luria-Bertani (LB) medium at 37°C with shaking. Cultures were diluted 1:1000 in fresh media in a volume of 200 *μ*L in a 96-well plate. Cell density was determined by incubating the plate at 37°C in a BioTek plate reader that shook the plate for 5 minutes before each read, every 20 minutes. Absorbance was read at 600 nm. The change in OD_600_ (ΔOD_600_) was calculated by subtracting the OD_600_ at Time = 0 from the OD_600_ at each selected time point. **(A)** Growth of single-effector deletion mutants in LB. **(B)** Growth of sequential-effector deletion mutants in LB. **(C)** Growth of multiple-effector deletion mutants in LB.
(TIF)

S1 Appendix Data for figures. (PZFX)
