# Supplementary figures and images for: Multiple Salmonella-pathogenicity island 2 effectors are required to facilitate bacterial establishment of its intracellular niche and virulence
Source: PLoS One. 2020 Jun 25;15(6):e0235020. doi: 10.1371/journal.pone.0235020 (PMC7316343; doi:10.1371/journal.pone.0235020)

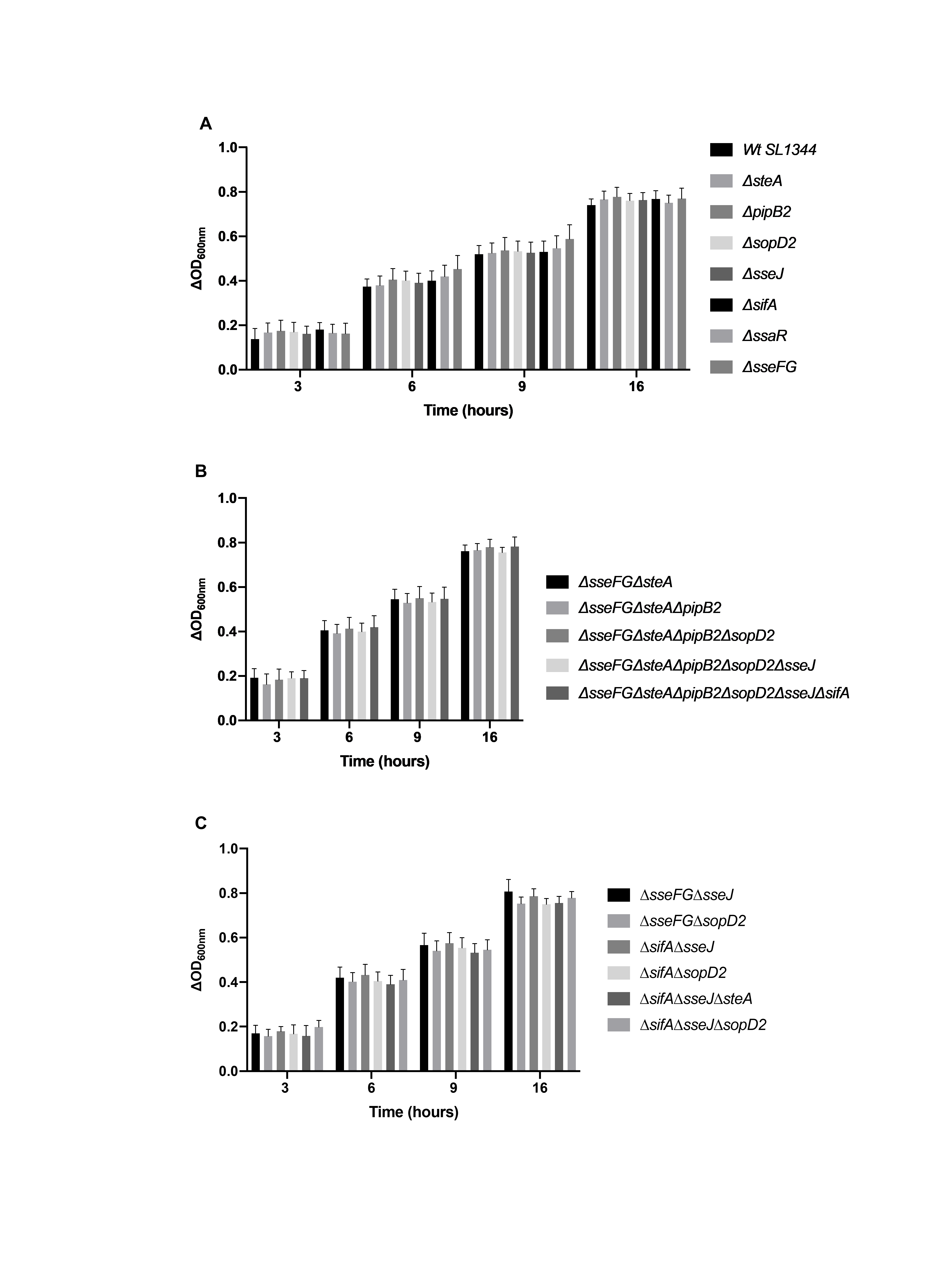

Supplement: S1 Fig — Single- and multiple-effector deletion mutants do not have impaired growth in LB liquid culture. 3 mL cultures of each strains were grown for 16–20 hours in Luria-Bertani (LB) medium at 37°C with shaking. Cultures were diluted 1:1000 in fresh media in a volume of 200 μL in a 96-well plate. Cell density was determined by incubating the plate at 37°C in a BioTek plate reader that shook the plate for 5 minutes before each read, every 20 minutes. Absorbance was read at 600 nm. The change in OD600 (ΔOD600) was calculated by subtracting the OD600 at Time = 0 from the OD600 at each selected time point. (A) Growth of single-effector deletion mutants in LB. (B) Growth of sequential-effector deletion mutants in LB. (C) Growth of multiple-effector deletion mutants in LB. (TIF) [file pone.0235020.s001.TIF]
